# Veillonella Bacteremia in a Patient With Metastatic Colorectal Carcinoma

**DOI:** 10.7759/cureus.41152

**Published:** 2023-06-29

**Authors:** Sailesh Karki, Arjun Mainali, Sagar Pandey, Navodita Uprety, Kalpana Panigrahi, Samaj Adhikari

**Affiliations:** 1 Internal Medicine, One Brooklyn Health-Interfaith Medical Center, Brooklyn, USA

**Keywords:** gut dysbiosis, colorectal cancer, gut microbiomes, bacteremia, veillonella

## Abstract

Colorectal carcinoma has increasingly been reported to be associated with gut microbial dysbiosis. *Bacteroides*, *Fusobacterium*, *Faecalibacterium*, *Blautia*, etc., are gut microbes commonly associated with colorectal carcinoma. Gut microbial dysregulation secondary to infectious, inflammatory, toxin exposure or change in dietary habits coupled with the disruption of the inner mucosal layer overlying the luminal epithelium is hypothesized as the inciting events leading to microbial invasion and subsequent tumorigenesis. Although the precise mechanism is unclear, disruption of normal host responses like inflammation, apoptosis, cellular proliferation, free radical injury, production of oncogenic toxins, etc., is postulated to play a role. We report a case of *Veillonella* *bacteremia* in a patient with metastatic colorectal carcinoma without a preceding history of periodontal disease. The patient was managed with ampicillin-sulbactam, which was followed by subsequent negative blood cultures. This case report signifies the association of gut microbiota like *Veillonella* with colorectal carcinoma and the importance of subsequent screening for colorectal cancer following *Veillonella bacteremia*.

## Introduction

*Veillonella* species are non-motile anaerobic gram-negative diplococci that constitute normal flora in the human mouth, gastrointestinal tract, and vagina [1]. *Veillonella atypica*, *Veillonella dispar*, and *Veillonella parvula* are the three major species that have been reported to cause human infection [2]. *Veillonella parvula* is the most frequently identified isolate [3]. *Veillonella* has been reported to cause severe infections, including endocarditis, discitis, psoas abscess, meningitis, and prosthetic joint infection [4,5]. *Veillonella* infection has been reported as a complication of endoscopy, colonoscopy, and salivary contamination with injection drug use [6,7]. The role of several gram-negative and anaerobic bacteria in tumorigenesis, tumor proliferation, and metastasis of colorectal cancer has been highlighted in the literature [1,8]. *Veillonella* species bacteremia in a patient with colorectal carcinoma is a unique occurrence that could support the potential role of the bacteria in the occurrence of malignancy. As per our knowledge, this is the first case of spontaneous *Veillonella* *bacteremia* in a patient with colorectal cancer.

## Case presentation

A 65-year-old male with no significant past medical history presented to the emergency room with complaints of generalized abdominal pain, bilateral leg swelling, and fatigue for one month. Abdominal pain was mild to moderate in intensity, dull aching, non-radiating, associated with abdominal distention, severe loss of appetite, unintentional weight loss, and constipation for the same duration. The last bowel movement was reported one week before the presentation. The patient also reported shortness of breath for one month with a functional capacity of < 1 block associated with orthopnea and a productive cough yielding a non-bloody, whitish sputum cough. In addition, the patient reported a history of subjective fever, undocumented, associated with chills a few days before presentation. The history of dysuria, urgency, frequency, headache, neck stiffness, or blurring of vision was negative. The patient denied any history of chest pain, palpitation, or syncopal episodes. No reported history of soft tissue infections was present. He had a significant smoking history of 30 pack years and endorsed smoking marijuana occasionally but denied alcohol intake or the use of other illicit drugs. The family history was negative for malignancy.

**Figure 1:**
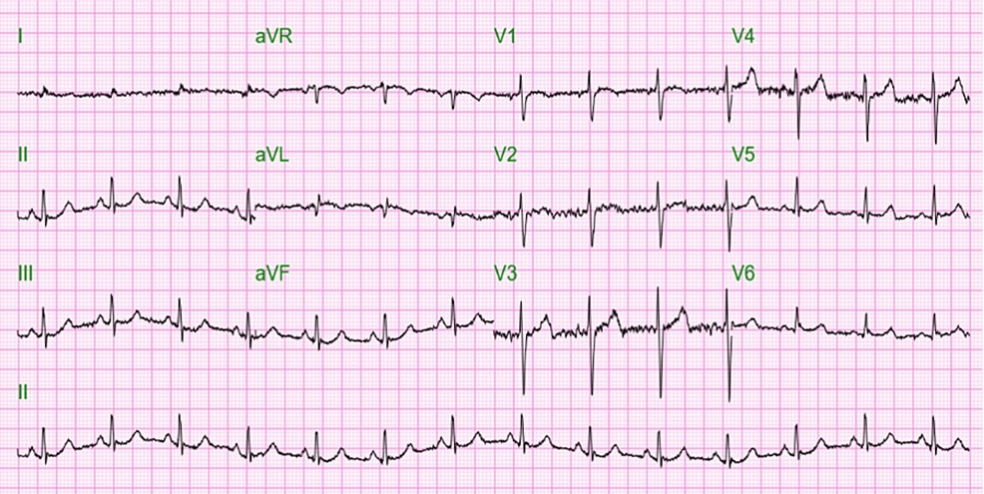
Electrocardiogram (EKG) at presentation showed normal sinus rhythm

**Figure 2:**
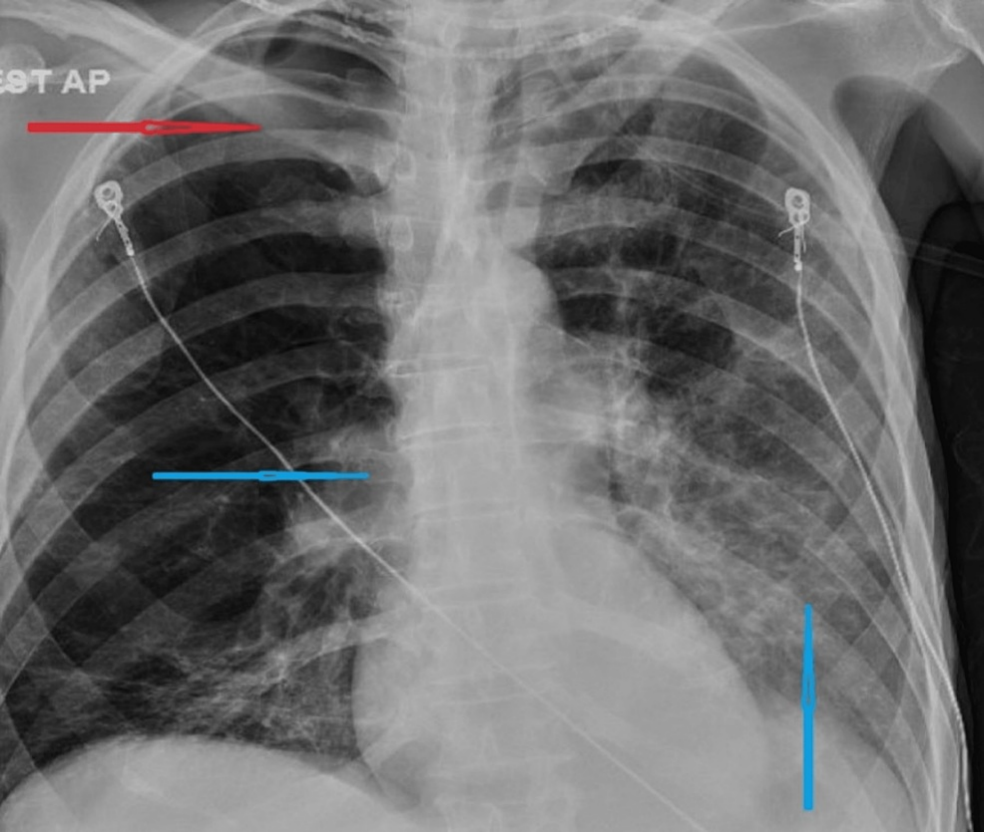
Chest X-ray at presentation showing left lower lobe and right infrahilar opacity (blue arrows) and right apical bullous change (red arrow)

Triage vitals showed a blood pressure of 100/66 mm Hg, a heart rate of 85 per minute, a temperature of 97.4 degrees Fahrenheit, a respiratory rate of 12 per minute, and an oxygen saturation of 100% in room air. The patient appeared ill-looking and cachectic, with bilateral pitting edema of the lower limbs. The abdominal examination showed generalized abdominal distension with mild non-localized tenderness but no rebound tenderness or rigidity. Bowel sounds were normoactive. Examination of the chest showed bilaterally decreased vesicular breath sounds, with bilateral wheezes and crepitations. Cardiovascular, neurological, and genitourinary examinations were within normal limits. The electrocardiogram at presentation showed normal sinus rhythm with a heart rate of 83 beats per minute (Figure 1). Chest X-ray was reported as left lower lobe and right infrahilar opacity concerning pneumonia with right apical bullous changes (Figure 2).

Laboratory findings were notable for microcytic anemia (mean corpuscular volume: 70.9 femtoliter) with no noted leukocytosis or lactic acidosis. Deranged liver function tests prompted testing for hepatitis viral serology, which was reported as normal. Pertinent laboratory parameters at presentation are depicted in Table 1.

**Table 1:** Laboratory parameters at the presentation AST: aspartate transaminase, ALT: alanine transaminase, ALP: alkaline phosphatase, INR: international normalized ratio, CEA: carcinoembryonic antigen, AFP: alpha fetoprotein, CA 19-9: cancer antigen 19-9, PSA: prostate-specific antigen, NA: not applicable

Laboratory parameter	Levels at presentation	Normal range
Hemoglobin	9.8	13-17 gm/dL
Hematocrit	32.4	39-53 %
White blood cells	8.5	4.5-11 10x3/uL
Platelets	360	130-400 10x3/uL
Blood urea nitrogen	59	7-25 mg/dL
Creatinine	1.2	0.7-1.3 mg/dL
Total protein	5.9	6.4-8.9 g/dL
Albumin	3.3	3.5-5.7 g/dL
Total bilirubin	4.4	0.3-1.0 mg/dL
Direct bilirubin	2.05	0.03-0.18 mg/dL
ALT	173	7-52 U/L
AST	116	13-39 U/L
ALP	942	34-104 U/L
Prothrombin time	14.6	9.8-13.4 sec
INR	1.22	0.85-1.15
Lactic acid	6.2	0.5-2.23 mmol/L
CEA	62.4	0.0-4.7 ng/ml
AFP	4.2	0.0-8.4 ng/ml
CA 19-9	4	0-35 U/ml
Total PSA	0.4	0-4 ng/ml
Free PSA	0.13	NA

**Figure 3:**
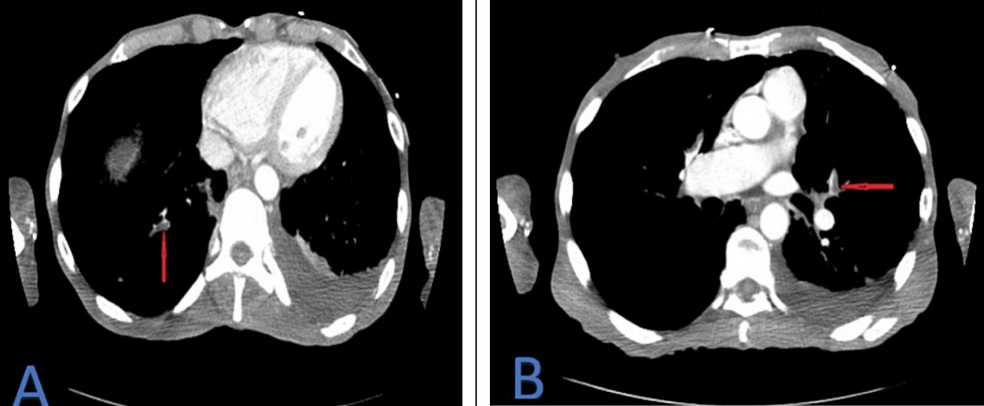
Computed tomography (CT) of the chest with contrast in the axial sections (A and B) with red arrows pointing towards filling defects in the subsegmental arteries in the bilateral lower lobes

**Figure 4:**
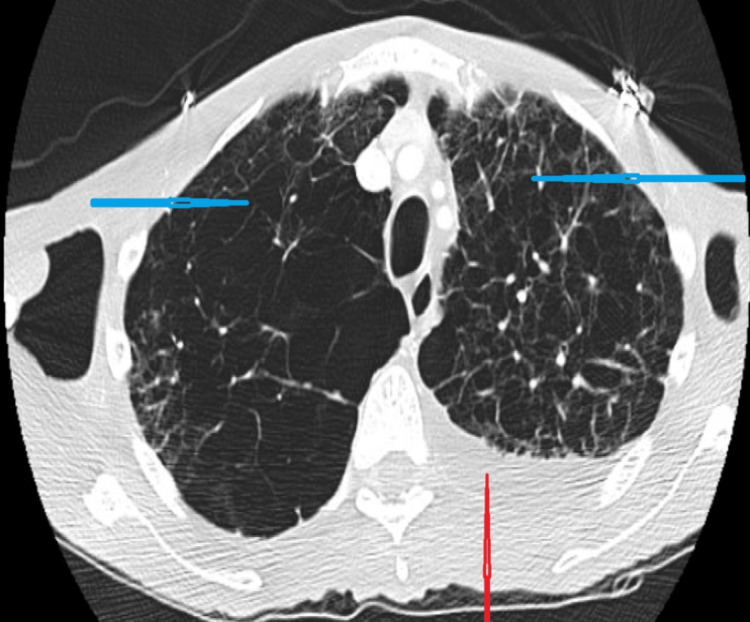
Computed tomography (CT) of the chest in axial section showing severe upper lobe emphysematous changes (blue arrows) and left pleural effusion (red arrow)

**Figure 5:**
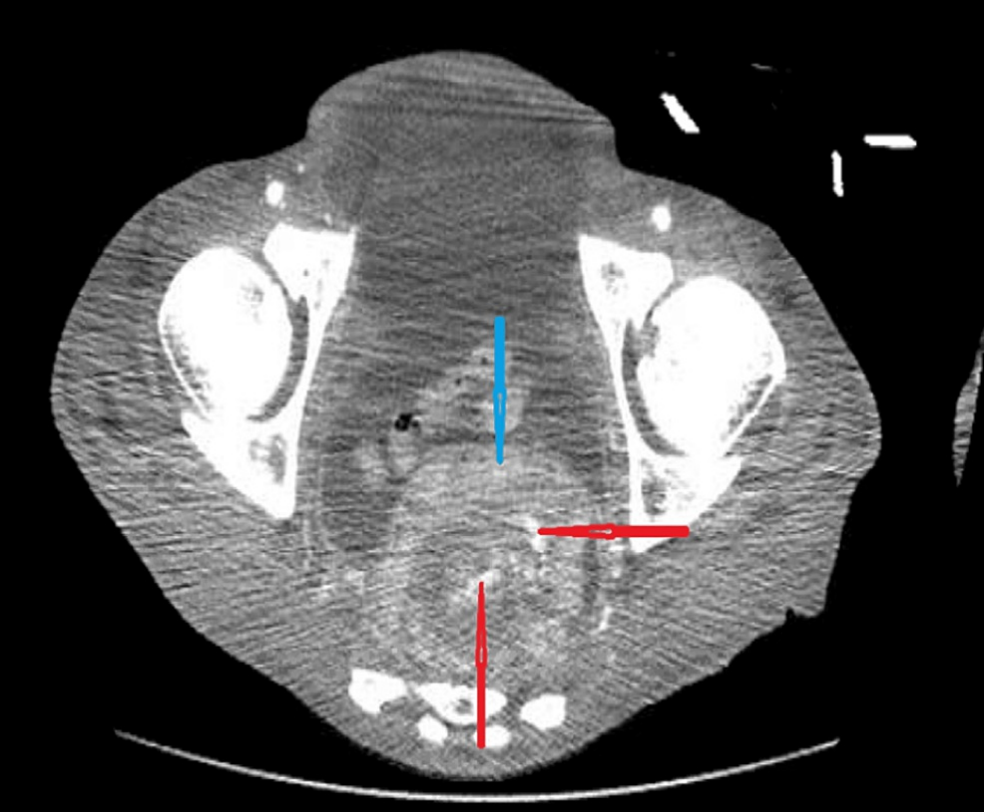
Computed tomography (CT) of the abdomen and pelvis in axial section shows abnormal moderate nodular rectosigmoid colonic wall thickening (blue arrow) with angiogenesis (red arrow)

**Figure 6:**
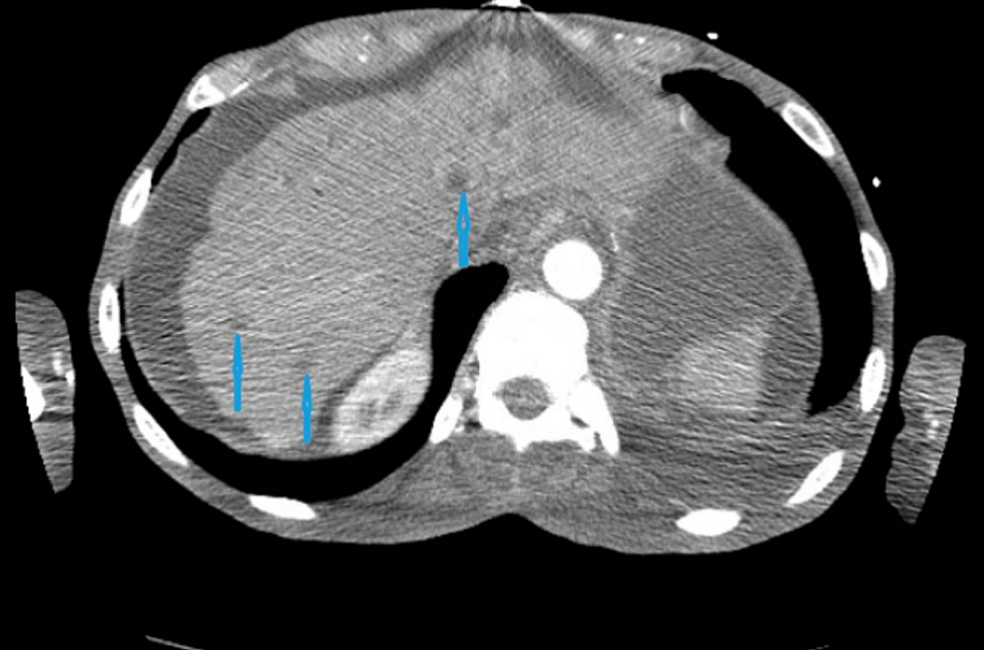
Computed tomography (CT) of the abdomen and pelvis in axial section shows multiple hypodensities in the liver parenchyma from metastatic lesions (blue arrows)

Computed tomography (CT) of the chest, abdomen, and pelvis with and without contrast revealed bilateral lower lobe pulmonary emboli (Figure 3), severe upper lobe emphysema (Figure 4), abnormal moderate nodular rectosigmoid colonic wall thickening with increased blood flow highly suspicious for colon carcinoma with tumor angiogenesis (Figure 5), ascites, and multifocal hypodensities in the liver parenchyma suggestive of metastases (Figure 6). Subsequently, an echocardiogram done to rule out right ventricular strain showed a normal left ventricular systolic fraction with an ejection fraction of 60%-65%, a mildly dilated right ventricle (pulmonary arterial systolic pressure, PASP: 55-60 mm Hg), mild pulmonic regurgitation, moderately severe tricuspid regurgitation, and a large pleural effusion. Venous doppler of the bilateral lower extremities showed no evidence of deep vein thrombosis. The patient was managed with ceftriaxone for pneumonia with a plan to de-escalate antibiotics following a blood culture and sensitivity results. A therapeutic dose of enoxaparin was started for pulmonary embolism as the patient was hemodynamically stable. 

A blood culture sent at the presentation isolated *Veillonella* species from anaerobic culture. Antibiotics were deescalated to ampicillin and sulbactam. Three sets of subsequent repeat blood cultures were negative for any growth. Although only a set of blood cultures positive for *Veillonella* species can be argued as a possible contaminant, the isolation of said organism in the setting of a patient with fever and an immunocompromised state from suspected metastatic adenocarcinoma of the gastrointestinal tract raises the probability of the culture result representing a true pathogen with a possible intra-abdominal source of infection.

Lastly, as a part of the workup for suspected intra-abdominal malignancy, tumor markers like alpha-fetoprotein, cancer antigen 19-9 (CA 19-9), and total/free prostate-specific antigen (PSA) were within the normal range with elevated carcinoembryonic antigen (CEA) levels (Table 1). The patient was planned for a colonoscopy with biopsy, but the procedure could not be done as the patient refused bowel preparation before the procedure. Diagnostic abdominal paracentesis showed hemorrhagic ascitic fluid (red blood cells, 1900 cells/uL) with normal white cell count and biochemical parameters like albumin, protein, amylase, glucose, lactate dehydrogenase, and lipase. Ascitic fluid cytology revealed malignant cells that were positive for Ber-EP4, Moc31, B72.3, CEA, and negative for B27.3 WT1 and Calretinin on immunohistochemical staining. Ber-Ep4 is found to be 80% sensitive and 94% specific for detecting metastatic adenocarcinoma in serous effusion. Furthermore, immunohistochemical staining positive for MOC31 and negative for D2-10/calretinin is reported to be 100% specific and 99% sensitive for differentiating malignant adenocarcinoma cells from reactive mesothelial cells [9]. With the intra-abdominal CT findings, elevated CEA, and malignant cells in ascites fluid cytology with the abovementioned immunohistochemical staining, the likelihood of primary colorectal cancer with hepatic metastasis was high. However, tissue diagnosis could not be obtained via colonoscopy; the patient later opted for do-not-resuscitate/do-not-intubate (DNR/DNI) with comfort care and eventually succumbed to death on day 13 of admission. The patient received a total of six days of ampicillin and sulbactam during her hospital stay. 

## Discussion

The human gastrointestinal tract has a plethora of microorganisms collectively called the gut microbiota, which play a vital role in host metabolism, immune regulation, and the prevention of colonization by pathogenic microorganisms. An intricate balance between the host gastrointestinal tract and the microbial ecosystem is maintained in a natural state. Gut dysbiosis, a term coined over a century ago, refers to the altered composition of the gut microbiota secondary to infectious, inflammatory, or toxin exposure or a change in dietary habits leading to a functional change in the microbial transcriptome, proteome, or metabolome [10]. The disruption of the inner mucosal layer overlying the luminal epithelium, facilitating direct interaction between mucosal epithelial cells and microbes, is postulated as one of the inciting events behind bacterial oncogenesis in the gut. While the precise mechanism is still uncertain, disruption of normal host responses like inflammation, apoptosis, and cellular proliferation, along with free radical injury, the production of oncogenic toxins, etc., are some of the postulated theories behind the phenomenon [11]. 

Colorectal carcinoma has been associated with an increased prevalence of *Bacteroides*, *Fusobacterium*, *Faecalibacterium*, and *Blautia* in the gut microbiome [8]. Bacteremia with *Streptococcus gallolyticus* and *Bacteroides* fragilis has been associated with a subsequent diagnosis of colorectal cancer in a retrospective study by Kwong et al. [12]. Exploiting the association, studies have explored the quantification of the associated fecal microbiome as an adjuvant modality for the detection of colorectal carcinoma [13]. *Veillonella bacteremia* in our patient with high suspicion of colorectal cancer could be attributed as a result of dysbiotic mucosa created by colorectal cancer, leading to the abnormal entry of the gut microbiome into the bloodstream. It further emphasizes the need for further research to understand the complex relationship between gut microbiota and colorectal neoplasms. Although reports of *Veillonella bacteremia* in a patient with bladder cancer have been documented in the literature [3], this is the first reported case of *Veillonella* in a patient with colorectal carcinoma with no history of periodontal disease. Colorectal tumor-associated increased production of interleukins (IL) like IL-23 and IL-17, along with a lack of barrier proteins and tight junction proteins with colorectal neoplasms, are hypothesized behind microbial invasion associated with colorectal neoplasms [12].

Due to the rarity of the presentation, the literature on appropriate antimicrobial treatment for *Velionella* is limited to a few reported cases [14]. A wide range of antibiotics has been used for treatment, which includes penicillin, metronidazole, cephalosporin, aminoglycosides, imipenem, clindamycin, doxycycline, erythromycin, and chloramphenicol [15]. In a review of literature related to *Velionella*, the most commonly reported infections associated with *Velionella* were bacteremia (37.5%), bone and joint infectious diseases (33.3%), and endocarditis (20.8%). In addition, the most commonly used antibiotics were penicillin (50%), followed by metronidazole (29.2%) and cephalosporins (20.8%), with combination antibiotic regimens being the most common strategy for treatment [15]. In this case, the patient was managed with intravenous ceftriaxone, which was later switched to ampicillin sulbactam following blood culture results. Antibiotics were discontinued after three negative blood culture results after six days of initiation. With the growing body of literature reporting the association of colorectal cancer with microbial dysbiosis, our case report highlights the probable association of yet another gut microbe, *Veillonella*, with colorectal cancer. 

## Conclusions

Colorectal carcinoma, with its enormous disease burden, is associated with gut microbial dysbiosis. *Veillonella bacteremia* is reported to be one of the gut microbes associated with colorectal carcinoma. The isolation of *Veillonella bacteremia* in a patient with colorectal carcinoma in this case report further signifies the validity of the association. Recognition of *Veillonella bacteremia* should therefore prompt clinicians to screen the patient for colorectal carcinoma as the next step in the workup.
